# 罕见*CRISPLD2-NRG1*融合突变晚期混合型非小细胞肺癌1例并文献复习

**DOI:** 10.3779/j.issn.1009-3419.2024.102.19

**Published:** 2024-05-20

**Authors:** CHEN Chunmei, YU Yang, HUANG Meijuan

**Affiliations:** ^1^610073 成都，成都市成飞医院呼吸与危重症医学科（陈春梅）; ^1^Respiratory and Critical Care Medicine, Chengfei Hospital, Chengdu 610073, China; ^2^610041 成都，四川大学华西医院胸部肿瘤科（喻杨，黄媚娟）; ^2^Thoracic Oncology Center, West China Hospital, Sichuan University, Chengdu 610041, China

**Keywords:** 肺肿瘤, 阿法替尼, CRISPLD2-NRG1融合突变, Lung neoplasms, Afatinib, CRISPLD2-NRG1 fusion mutation

## Abstract

肺癌是中国发病率和死亡率最高的恶性肿瘤。非小细胞肺癌（non-small cell lung cancer, NSCLC）占全部肺癌的80%以上，NSCLC的基因突变概率高，并且种类繁多。随着基因检测技术的进步，越来越多的罕见融合基因变异被检测出来。神经调节蛋白1（neuregulin 1, NRG1）可促使人表皮生长因子受体3（human epidermal growth factor receptor 3, Her3/ErbB3）介导的通路激活，从而导致肿瘤形成。本文报道了1例罕见CRISPLD2-NRG1融合突变的晚期混合型NSCLC颅内转移的患者，接受阿法替尼治疗1个月后头部磁共振成像（magnetic resonance imaging, MRI）显示颅内病灶明显缩小，患者对阿法替尼治疗反应良好。同时，我们对以往报道的NRG1基因融合突变的NSCLC病例进行总结，以供临床借鉴。

## Abstract (en)

Lung cancer is the most common malignant disease and the leading cause of cancer death in China. Non-small cell lung cancer (NSCLC) accounts for over 80% of all lung cancers, and the probability of NSCLC gene mutations is high, with a wide variety of types. With the development of next-generation sequencing (NGS) detection technology, more and more patients with rare fusion gene mutations are detected. Neuregulin 1 (NRG1) gene is a rare oncogenic driver that can lead to activation of human epidermal growth factor receptor 3 (Her3/ErbB3) mediated pathway, resulting in tumor formation. In this article, we reported a case of mixed NSCLC with CRISPLD2-NRG1 fusion detected by RNA-based NGS, who responsed to Afatinib well after 1 month of treatment, and magnetic resonance imaging (MRI) showed shrinkage of intracranial lesions. Meanwhile, we also compiled previously reported NSCLC patients with NRG1 rare gene fusion mutation, in order to provide effective references for clinical diagnosis and treatment.

神经调节蛋白1（neuregulin 1, NRG1）是介导细胞间相互作用的信号蛋白，参与组织细胞的发育和成熟。NRG1基因主要与细胞表面的人表皮生长因子受体3（human epidermal growth factor receptor 3, Her3/ErbB3）结合，激活ErbB3，而后ErbB3与ErbB2形成异二聚体，进而激活下游的丝裂原活化蛋白激酶（mitogen-activated protein kinase, MAPK）及哺乳动物雷帕霉素靶蛋白（mammalian target of rapamycin, mTOR）等信号通路，导致细胞增殖分化，从而促进肿瘤的形成和生长^[1,2]^。虽然NRG1融合突变最早于1999年在乳腺癌细胞系中被描述^[3]^，但直到2014年才首次观察到在肺癌中也携带有这种基因变异^[4]^。本文报道了1例罕见的CRISPLD2-NRG1融合突变晚期混合型NSCLC病例，并进行了文献复习。

## 1 病例资料

患者，男性，67岁，因“左肺癌术后反复右侧肢体乏力1年，再发15天”就诊。2年前胸部计算机断层扫描（computed tomography, CT）发现左肺上叶结节，左肺门、纵隔淋巴结增大。2021年6月11日行“左上肺楔形切除术+纵隔淋巴清扫”，术后病理：左上肺混合癌（肉瘤样癌+腺癌），分期为pT2aN0M0 IB期，程序性死亡配体1（programmed cell death ligand 1, PD-L1）-22C3 70%。基因检测结果均未见异常突变。术后患者拒绝化疗。2022年3月首次出现右侧肢体乏力，肌力III级。头部磁共振成像（magnetic resonance imaging, MRI）（2022年4月15日）检查发现左侧顶叶占位，病灶周围水肿明显，考虑为转移瘤（图1A）。行颈胸腹部增强CT、单光子发射计算机断层成像术（single-photon emission computed tomography, SPECT）等检查，未发现其他部位转移病灶。2022年4月16日行“左侧顶叶侧脑室占位切除术+窦修补术+脑脊液漏修补术”，术后病理：腺癌，免疫组化确认符合肺来源低分化癌（含腺癌成分）转移。血液+左额叶组织配套标本下一代测序（next-generation sequencing, NGS）检测（2022年5月6日）：RAD51D p.K91Ifs*13丰度50%。术后患者右侧肢体乏力有缓解，肌力IV级。术后第37天复查头部增强MRI（2022年5月23日）示：左侧顶叶术后残腔边缘多发强化壁结节，周围见片状水肿信号，左侧侧脑室后角受压（图1B）。基于患者肺组织PD-L1高表达，于2022年5月26日开始予以帕博利珠单抗联合贝伐珠单抗治疗共6个疗程（图1C）。进一步行头部正电子发射型磁共振成像（positron emission tomography/magnetic resonance, PET/MR）（2022年6月7日）示：左侧顶叶残腔腔壁多处异常信号伴代谢增高，为肿瘤复发可能性大；同侧额顶叶脑实质水肿。2022年6月13日至15日行左侧顶叶病灶放疗：6 Gy/2 Gy/3 f。最佳疗效评估为部分缓解（partial response, PR）。6个周期后患者出现丙氨酸氨基转移酶升高，最高204 IU/L，根据不良事件通用术语评价标准5.0版（Common Terminology Criteria for Adverse Events 5.0, CTCAE 5.0）考虑为免疫治疗相关性肝炎，CTACE 3级肝功能损伤，暂停免疫治疗。2023年1月28日患者再次出现右侧肢体乏力，肌力III级。头部增强MRI（2023年2月1日）示：左侧顶叶术后残腔边缘多发强化结节（图1D）。于2023年2月23日行“左侧顶枕叶、胼胝体、侧脑室占位切除术+窦损伤修补术+脑脊液侧漏修补术”手术。术后病理：腺癌，免疫组化确认肺来源。2023年3月18日患者右侧肢体乏力加重，肌力II级，伴有吐词不清。头部增强MRI（2023年3月29日）示左侧顶叶术后残腔边缘及左侧脑室前角边缘、透明隔区多发明显强化结节（图1E）。脑组织标本RNA测序检测结果示：CRISPLD2-NRG1融合，丰度1.85%。于2023年4月11日开始口服阿法替尼40 mg qd作为二线治疗。2023年4月11日至24日行全脑放疗（ whole brain radiation therapy, WBRT）30 Gy/3 Gy/10 f。1个月后患者门诊行疗效评估，右侧肢体肌力IV级，吐词不清较前明显改善。复查头部MRI增强扫描后根据实体瘤疗效评价标准（Response Evaluation Criteria in Solid Tumor, RECIST）1.1版评价为PR（图1F）。门诊随访至2023年9月（图1G），患者一般状况良好，头部MRI检查提示病灶明显缩小，无进展生存期（progression-free survival, PFS）为6个月。2023年10月患者死于侵袭性肺曲霉菌病。

**图 1:**
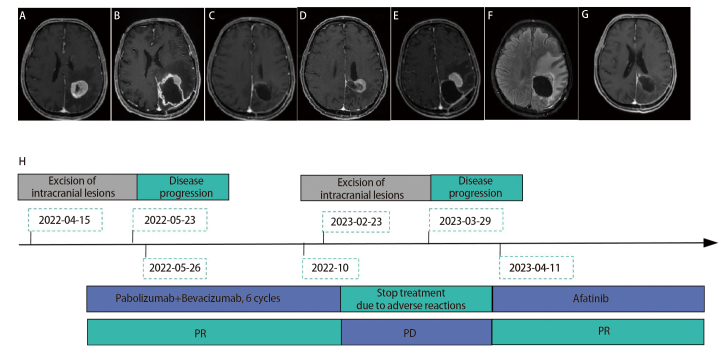
患者治疗前后头MRI的改变。A：2022年4月15日首次发现颅内转移病灶；B：2022年5月23日颅内病灶手术后复发；C：2022年5月26日帕博利珠单抗联合贝伐珠单抗治疗6个疗程后颅内病灶缩小；D：2023年2月1日颅内病灶再复发；E：2023年3月29日颅内病灶第二次手术后复发；F：2023年5月11日阿法替尼治疗1个月后颅内病灶缩小；G：2023年8月10日口服阿法替尼4个月后；H：2022年4月15日至2023年10月29日患者病情演变及治疗流程图。

## 2 讨论

NRG1是表皮生长因子（epidermal growth factor, EGF）配体家族的一员，几乎所有的NRG1都有EGF样结构域。其EGF样结构域以自分泌或旁分泌的方式与ErbB3结合，导致ErbB2-ErbB3异源二聚体的形成与活化，然后通过磷脂酰肌醇3-激酶/蛋白激酶B（phosphatidylinositol 3-kinase/protein kinase B, PI3K/Akt）和肾素-血管紧张素系统（renin angiotensin system, RAS）/MAPK途径激活下游信号传导，促进细胞增殖分化，从而促进肿瘤的形成和发展^[4,5]^。

两项大规模癌症驱动基因的数据分析^[6,7]^显示，约0.3%的NSCLC中存在NRG1基因融合突变。NRG1基因的融合伴侣异质性较大，目前已报道的有CD74、SLC3A2、RBPMS、SDC4、VAMP2、FOXA1等。复旦大学陈海泉研究团队^[8]^对1681例中国肺腺癌患者进行了分析，NRG1融合发生率为0.36%（6/1681），融合伴侣分别为CD74（4例）、RBPMS（1例）和ITGB1（1例）。NRG1融合变异与侵袭性黏液型肺腺癌高度相关，并且是潜在可治疗的致癌驱动改变，常发生于不吸烟的女性肺腺癌患者，这部分患者的其他肿瘤已知致癌基因突变大部分呈阴性^[9,10]^。本例患者为男性，无吸烟史，肺内病灶和颅内转移病灶的表皮生长因子受体（epidermal growth factor receptor, EGFR）、间变性淋巴瘤激酶（anaplastic lymphoma kinase, ALK）、RAS、Kirsten大鼠肉瘤病毒癌基因同源物（Kirsten rats arcomaviral oncogene homolo, KRAS）表达均为阴性。研究^[11,12]^发现，CD74-NRG1融合突变导致NRG1 III-β3亚型的EGF样结构域过表达，NRG1 III-β3作为ErbB3的配体，诱导其磷酸化并随后激活下游PI3K-Akt通路，导致肿瘤发生。因此，我们推测CRISPLD2-NRG1融合突变可能通过同样的路径，促进肿瘤转移。

融合基因检测的金标准为基于DNA的荧光原位杂交检测（fluorescence in situ hybridization, FISH），然而由于NSCLC融合基因数目较多，具有高通量优势的NGS可以同时检测多个基因融合变异，因此被广泛应用到融合基因检测中。NGS检测对象为DNA和RNA，文献报道的NRG1融合变异都是采用RNA-based NGS检测出来的，这可能与基因存在大量重复序列的内含子和基因在DNA层面融合丰度低等因素有关。在RNA水平上检测融合基因比DNA水平更敏感^[9,10]^。本例患者第一次颅内病灶术后标本及血液基因检测发现RAD51D基因突变，RAD51D基因属于同源重组修复（homologous recombination repair, HRR）通路基因，通过与多聚ADP核糖聚合酶（poly ADP-ribose polymerase, PARP）的合成致死效应从而抑制肿瘤细胞的发生。RAD51D基因突变的患者可能从PARP抑制剂，如奥拉帕利、尼拉帕利等治疗中获益^[13,14]^。但使用该类药物治疗携带RAD51D基因的肺癌患者属于跨适应证用药。本例患者肺组织PD-L1高表达，因此后续给予了帕博利珠单抗治疗，最佳疗效PR，但出现了CTACE 3级肝功能损伤。患者无肺癌家族史，在第二次颅内病灶手术切除后，我们对手术标本进行了RNA-based NGS检测，发现了CRISPLD2-NRG1基因融合突变。

NGR1基因融合突变的患者通常对标准化疗或免疫检查点抑制剂治疗反应不佳。根据致癌机制，NRG1基因融合及其下游信号通路是治疗干预的可行靶点^[15]^。ErbB的靶向治疗包括抗ErbB3抗体Lumretuzumab^[16]^、Patritumab、Gsk2849330、双特异性人源化单克隆抗体Zenocuzumab^[17]^以及小分子酪氨酸激酶抑制剂（tyrosine kinase inhibitors, TKIs）药物，比如阿法替尼、拉帕替尼、他雷替尼等。在包括拉帕替尼和阿法替尼在内的肿瘤模型中，NRG1基因融合突变的肿瘤对EGFR、ErbB2和ErbB3抑制剂均有应答，其中阿法替尼的疗效最为显著^[18,19]^。既往报道的11例NRG1融合突变肺癌患者的病例的临床资料见表1^[20⇓⇓⇓⇓-25]^，以不吸烟人群为主（不吸烟:吸烟=10:1），女性居多（女:男=7:4），年龄最小34岁，最大80岁，所有患者病理类型都是肺腺癌，对化疗不敏感。均采用RNA-based NGS检测方法。融合突变类型中有3例为SDC4-NRG1，1例SLC3A2-NRG1，1例未提及融合伴侣，其余均为CD74-NRG1。治疗和转归方面，阿法替尼作为一线治疗5例，二线治疗3例，多线后治疗3例。阿法替尼的客观疗效评价为：客观缓解率（objective response rate, ORR）为90.9%（10/11），疾病控制率（disease control rate, DCR）为100.0%。1例疗效评估为病情稳定（stable disease, SD）的患者有20^+^包/年的吸烟史，发现SDC4-NRG1基因融合突变后给予阿法替尼治疗，SD维持4个月后疾病进展（progressive disease, PD）。另有1例伴有ErbB2 20外显子插入突变，同时给予了阿法替尼和吡格替尼联合WBRT治疗，疗效为PR，未报道PD或死亡。11例病例中仅有6例患者报道了PD时间，2例报道了死亡，因此无法计算平均PFS和总生存期（overal survival, OS）。

**表 1:** 罕见NRG1融合患者的临床特征和TKIs疗效

Reference	Type of NRG1 fusion	Age (yr)	Gender	Smoking status	Histology	Stage	Treatment	Line	ORR	PFS (mon)
Jones et al.^[20]^	SDC4-NRG1	43	Female	Never	ADC	NA	Afatinib	2	PR	12
Gay et al.^[21]^	SLC3A2-NRG1	42	Female	Never	ADC	IV	Afatinib	2	PR	12
	DC74-NRG1	62	Female	Never	ADC	IV	Afatinib	1	PR	10
Cheema et al.^[22]^	CD74-NRG1	62	Male	Never	ADC	IV	Afatinib	2	PR	6.2
Wu et al.^[23]^	CD74-NRG1	80	Male	Never	ADC	IV	Afatinib	1	PR	8
Chen et al.^[24]^	CD74-NRG1	62	Male	Never	ADC	IV	Afatinib+ Pyrotinib+ WBRT	1	PR	NA
Cadranel et al.^[25]^	NA	62	Female	Never	ADC	IV	Afatinib	15	PR	4
	CD74-NRG1	62	Female	Never	ADC	IV	Afatinib	5	PR	11
	SDC4-NRG1	68	Male	Yes	ADC	NA	Afatinib	1	SD	4
	CD74-NRG1	43	Female	Never	ADC	IV	Afatinib	5	PR	24
	SDC4-NRG1	34	Female	Never	ADC	IIIB	Afatinib	1	PR	5

NRG1: neuregulin 1; TKIs: tyrosine kinase inhibitors; ADC: adenocarcinoma; ORR: objective response rate; PFS: progression-free survival; NA: not available; WBRT: whole brain radiation therapy; SD: stable disease.

阿法替尼为不可逆的泛EGFR-TKIs，可能是CRISPLD2-NRG1基因融合突变肿瘤的潜在治疗选择。Tamiya等^[26]^研究发现，阿法替尼的脑脊液浓度为（3.16±1.95）ng/mL，血脑屏障渗透率为2.45%±2.91%。研究^[27]^证实，EGFR-TKIs联合放疗具有协同作用，疗效优于单纯EGFR-TKIs或放疗。放疗与EGFR-TKIs之间的相互作用机制包括：放射对EGFR-TKIs的增敏作用和辐射对血脑屏障的开放作用^[28,29]^。EGFR-TKIs抑制肿瘤细胞放疗后的DNA损伤修复，增加对放疗敏感的G_1_、G_2_和M期的细胞比例，减少对放疗不敏感的S期细胞比例。另外，脑照射和脑转移会破坏血脑屏障的完整性，增加血脑屏障的通透性，导致EGFR-TKIs的入脑浓度增加^[28⇓⇓⇓-32]^。Zhuang等^[33]^的研究发现，有颅内转移的肺腺癌患者WBRT和WBRT同时联合厄洛替尼的ORR分别为54.84%和95.65%，中位OS分别为8.9和10.7个月，并且多因素分析表明，厄洛替尼是延长OS的重要预后因素。2023年4月患者基因检测发现CRISPLD2-NRG1基因融合突变后，我们选择了阿法替尼同时联合WBRT进行治疗。该患者在服药2周后出现了CTACE 3级的腹泻和口腔黏膜溃疡，根据阿法替尼不同程度药物相关不良事件的建议给药量，停药5 d后从20 mg/d开始逐渐增加剂量至40 mg/d，过程顺利。RECIST疗效评价为PR。

美国国立综合癌症网络（National Comprehensive Cancer Network, NCCN）公布的NSCLC临床实践指南指出，在广泛组合的检测中未发现驱动基因的患者（尤其是非吸烟者）需考虑采用RNA-based NGS检测^[34]^。我国于2023年11月发布的《基于RNA-based NGS检测非小细胞肺癌融合基因临床实践中国专家共识》对RNA-based NGS检测融合基因的应用时机等进行了详尽阐述，以最大程度地发现融合基因变异^[35]^。这篇报道中，我们通过RNA-based NGS检测呈现了1例罕见CRISPLD2-NRG1融合突变晚期的NSCLC病例，患者二线接受阿法替尼治疗后出现临床症状减轻，在死于侵袭性肺曲霉菌病前1个月颅内病灶仍控制良好。目前有关NRG1融合突变肺癌的流行病学、生物学和侵袭性尚无大规模研究数据，而阿法替尼对NRG1融合类型的疗效及治疗机制也有待于进一步明确。但鉴于此类基因突变患者数量少，因此单个的病例报告将有助于为临床治疗提供参考。
